# Classification and Parameter Selection for Damage Characterization in CFRP Composite Materials Using Acoustic Emission and Multivariate Statistics

**DOI:** 10.3390/ma19102091

**Published:** 2026-05-16

**Authors:** David Amoateng-Mensah, Richard Dela Amevorku, Pusan Dhar, Tanzila B. Minhaj, Mannur J. Sundaresan

**Affiliations:** Department of Mechanical Engineering, North Carolina A & T State University, 1601 E. Market Street, Greensboro, NC 27411, USA; rdamevorku@aggies.ncat.edu (R.D.A.); pdhar@aggies.ncat.edu (P.D.); tbminhaj@aggies.ncat.edu (T.B.M.); mannur@ncat.edu (M.J.S.)

**Keywords:** Acoustic Emission (AE), damage mechanisms in thermoset CFRP, carbon fiber composites, machine learning (ML), deep learning (DL), multivariate logistic regression

## Abstract

Accurate damage characterization in thermoset Carbon Fiber-Reinforced Polymer (CFRP) composites using Acoustic Emission (AE) requires statistically robust and interpretable models. This study employs multinomial logistic regression with forward selection and Type III analysis to identify the minimal set of AE parameters necessary for classifying damage mechanisms (fiber breaks, delamination, matrix cracks) in quasi-isotropic thermoset CFRP laminates under synchronously recorded load conditions. Starting from 18 conventional time- and frequency-domain descriptors, forward selection yielded seven candidate predictors. However, Type III analysis revealed that only four parameters, Load, Initiation Frequency, Amplitude, and Average Frequency, provide unique, statistically significant contributions (*p* < 0.05). The remaining predictors became redundant once these four were included. Machine learning and deep learning models trained on this minimal feature set achieved validation accuracies up to 98.7% on external specimens. High-frequency components (>1 MHz), as recorded at the sensor location after propagation and sensor convolution, were associated with fiber break events at elevated loads, while delamination events exhibited higher amplitude and lower-frequency content (<200 kHz) compared to matrix crack events. These observed frequency ranges reflect the combined effects of source mechanisms, guided wave dispersion in the 2.4 mm thick laminate, PWAS sensor response, and HDT-based hit segmentation, and are consistent with established AE damage signatures in literature. The results indicate that this four-parameter set is sufficient to classify the labeled AE waveform classes under monotonic tensile loading of quasi-isotropic [45/90/−45/0]_2s_ laminates, achieving 98.7% agreement with reference labels assigned via waveform morphology and spectral analysis. The proposed approach reduces computational overhead and enhances interpretability for structural health monitoring applications, pending validation across broader material systems and loading scenarios. A limitation of this study is that reference labels were assigned using waveform morphology and spectral analysis, lacking independent physical validation (e.g., microscopy).

## 1. Introduction

Carbon Fiber-Reinforced Polymer (CFRP) composites are increasingly being used in safety and performance-critical structures because of their high stiffness, strength and corrosion resistance. However, these advantages come with their own intrinsically multiscale damage evolution that includes matrix cracking, fiber breaks and delamination. These damage modes start early in the loading cycle and accumulate in an uneven fashion while remaining relatively invisible to surface inspection techniques until substantial fatigue and strength loss or unstable failure occurs. Acoustic Emission (AE) provides in situ and real-time sensing modality for CFRP composites because of its ability to capture the transient stress wave activities produced by irreversible micro-structure damage. This enables continuous monitoring of structures during service. AE can detect damage onset and track damage progression under quasi-static and fatigue loading conditions, and hence it has the potential for monitoring the structural health (SHM) of composite structures in service [1]. AE technique is a passive mode of damage monitoring compared to thermographic techniques that can either be active or passive depending on the application [2,3]. In previous research, the emphasis in CFRP studies was on correlating measured signal attributes with the corresponding underlying fracture processes. Early works by Berthelot et al. linked AE activity to defined fracture processes in carbon epoxy composites, establishing a foundation for interpreting AE trends during progressive failure [4]. Further studies explored frequency-related descriptors to discriminate fracture mechanisms including real-time characterization for different fracture sources [5] and fracture mechanism characterization in cross-ply carbon fiber composites using AE analysis [6]. Additional studies in CFRP have further strengthened the experimental basis for mapping AE features to damage modes and their chronology with varying layups and stress concentrators [7]. Finite element methods have also been used to model and distinguish between the three main failure modes, that is, matrix cracks, delamination and fiber breaks, based on their respective frequency components [8]. In addition, there are further studies that utilize various machine learning algorithms for damage detection in these materials [9,10,11].

Despite the diversity in literature attempting to deploy SHM particularly for large complex CFRP structures, parameter-only AE characterization is often used. In this method, each detected hit is summarized by a compact vector of time-domain and scalar descriptors such as amplitude, risetime, duration and other related derived indices. This method is convenient because of the reduction in data volume and the support for real-time processing of AE events that align with what most commercial AE systems read and export [12]. Parameter-only analysis must also consider the diverse nature of AE signals and the variability introduced by the guided wave propagation effects such as attenuation and dispersion that distort received AE signals especially in plate-like composite structures [13]. In addition, CFRP damage modes are diverse and can occur concurrently, making parameter-based signatures overlap, with a single AE hit consistent with more than one failure mode that reduces separability in parameter space [14]. These challenges motivate statistical modeling and damage characterization methods that utilize only AE parameters instead of the full waveform analysis using multivariate methods, clustering and other classifiers such as Principal Component Analysis (PCA) to improve accurate detection and classification when multiple damage mechanisms are present [15,16].

Recent advances in CFRP damage identification have explored sophisticated deep learning architectures beyond conventional supervised classification [17]. Graph neural networks (GNNs) have been applied to localize structural damage on wind turbine blades with high accuracy by converting AE signals into graph structures and aggregating neighboring node information [18]. There have been other applications of unsupervised GNNs to identify sandstone weathering levels from AE statistical features, outperforming alternative methods across four evaluation criteria [19]. Quang-Hung et al. evaluated GNNs for supervised classification of noisy acoustic signals from hydroelectric generators, comparing performance against Convolutional Neural Networks (CNNs) [20]. These studies demonstrate GNNs’ versatility across various sectors, though full performance metrics vary by application. In addition, GNNs require multi-sensor spatial data and are sensitive to sensor placement. Bayesian approaches offer probabilistic damage classification with uncertainty quantification, which is particularly valuable for risk-informed structural integrity assessment. Junfang Wang et al. developed a probabilistic rail condition assessment framework [21], whilst Masoud et al. integrated AE monitoring with recursive Bayesian updating for fatigue crack growth prediction [22]. More recent studies applied nonparametric Bayesian methods for automated event identification [23]. The primary strength of this method lies in uncertainty quantification and integration of multiple information sources, though applications remain largely experimental or case-study-based. These methods involve substantial computational overhead for posterior sampling. Variational Autoencoder–Convolutional Neural Network (VAE-CNN) architectures have demonstrated promising results for AE signal classification even with small training datasets, addressing a common limitation in composite testing where labeled damage events are scarce [24]. VAE-CNN models, while data-efficient, sacrifice interpretability due to latent space representations that lack direct physical meaning. In contrast, the logistic regression approach presented in this study prioritizes statistical interpretability and physical transparency. Each retained predictor can be directly linked to damage physics, and *p*-values provide formal significance testing. This is critical for industrial SHM deployment where model decisions must be explainable to certification authorities and maintenance personnel. The trade-off is that our method requires synchronous load recording and assumes parametric linearity in the logit space, whereas deep learning methods can capture arbitrary nonlinear relationships.

Logistic regression is a fundamental multivariate approach to modeling a dichotomous outcome in terms of several predictors and allows for estimation of adjusted associations and multivariate predictions within the framework of probability. It comprises fitting a sigmoid curve to data, mapping inputs to corresponding probabilities between 0 and 1. Logistic regression as a statistical method has been employed in many previous studies. R. Vidya et al. [25] used logistic regression for crack classification and damage assessment in reinforced concrete structures focused on distinguishing tensile and shear cracks using AE waveform parameters. Logistic regression was also used to classify AE signals from wear mechanisms in joint conditions, thus achieving high classification potential [26]. Jihong Yan et al. [27] utilized the technique for performance degradation assessment in industrial systems. The method’s strength lies in its ability to process non-stationary signals, extract critical features and provide probabilistic assessments of system conditions.

While previous studies have established that amplitude and frequency-related parameters are important discriminators for AE-based damage classification in composites [4,5,6,14], these findings have been based primarily on empirical observation, unsupervised clustering, or qualitative waveform analysis. The specific contribution of this study is threefold:

Formal statistical quantification of feature importance using multivariate forward selection and Type 3 redundancy analysis, providing *p*-values, effect sizes, and a principled redundancy assessment that goes beyond correlation-based or tree-based feature importance rankings commonly used in ML studies.

Evidence that, among the full set of conventional AE parameters, only four (Load, Initiation Frequency, Amplitude, Average Frequency) provide non-redundant discriminative information, with the remaining parameters offering no unique predictive contribution once these four are included.

Validation that this minimal parameter set maintains high classification accuracy (up to 98.7%) across multiple ML and DL architectures on data from a second specimen of the same layup, labeled using identical criteria, confirming practical sufficiency within the scope of the labeling methodology employed. However, this does not provide a substitute for independent physical validation.

This approach bridges the gap between traditional AE practices where parameter selection is often ad hoc and rigorous statistical inference, offering a reproducible framework that can be applied to other material systems.

## 2. Background

### 2.1. AE Parameters

**Figure 1:**
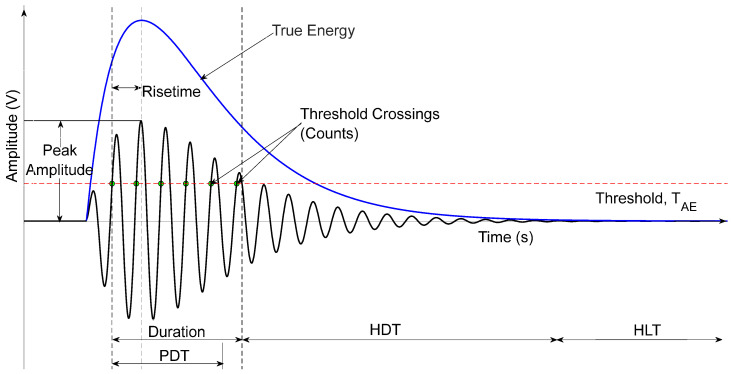
Figure illustrating sample AE parameters [10].

The following AE parameters, listed and explained below, were used as the AE Parameters in the logistic regression model. A sample of these parameters is illustrated below in Figure 1.

Risetime—This is defined as the time interval between the first threshold crossing and the peak amplitude of the AE signal. This parameter provides a measure of the rate of crack propagation. A short risetime indicates a sudden release of energy, usually indicative of brittle failure, whilst a longer risetime suggests a more gradual crack growth rate [28].

Counts to peak—This refers to the number of threshold crossings that occur between the first threshold crossing and the signal’s peak amplitude.

Counts—The counts parameter represents the number of times the AE signal crosses the set threshold during the signal duration. High counts indicate the presence of microstructural activity such as multiple crack growth events [29].

Energy—This is the sum of the integral of the squared voltage over the duration of the AE event, which is usually measured in electron volts (eV). It correlates with the amount of energy released by the crack source and gives a key indicator of the severity of the damage mechanism [30].

Duration—Duration is measured as the time between the first and last threshold crossing of the received AE signals. It reflects how long the source remains active and is influenced by wave propagation effects such as reflections and attenuation [28].

Amplitude—This is defined as the maximum voltage level recorded usually measured in volts or its equivalent units. It is proportional to the stress wave’s peak displacement providing a measure of the source’s strength [29].

ASL (Average Signal Level)—ASL is the root mean square (RMS) value of the signal voltage over a defined time window. It represents the overall signal intensity and is useful for monitoring background noise and other related emissions [30].

Threshold—This is defined as the minimum voltage level that must be exceeded for a signal to be recorded as an AE event. It must be set appropriately such that other electrical and ambient mechanical noise is eliminated whilst ensuring that genuine AE events are detected.

Average Frequency—This is a calculated parameter that is determined by dividing the total counts by the duration of the hit. This is used as a simplified estimation of the spectral content in a signal without performing a full Fourier Transform [31].

Root Mean Square (RMS)—The RMS voltage is the measure of the continuous AE signal intensity calculated over a specific time window, and it is sensitive to continuous emission sources instead of discrete bursts [32].

Reverberation Frequency—This is defined as the frequency of the signal after the peak amplitude has been recorded. It is calculated by dividing the counts after the peak amplitude by the time difference between the peak amplitude and the end of the last threshold crossing [33].

Initiation Frequency—This is the average frequency of the signal before the peak amplitude used to characterize the initial stage of the source activation [33].

Signal Strength—Signal Strength is defined as the area under the envelope of the rectified linear voltage time signal.

Absolute Energy—This is the true energy measure calculated from the integral of the squared voltage divided by the input impedance. It provides a standardized value that allows for comparison across different testing systems [31] and a differentiator between various wave propagation modes [34].

### 2.2. Damage Modes

**Figure 2:**
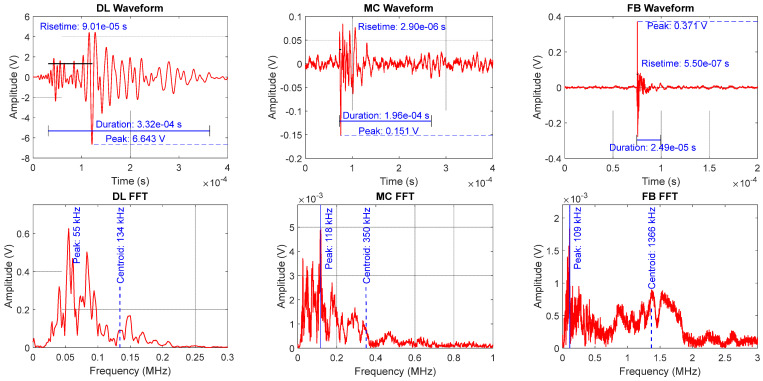
Representative waveforms and Fast Fourier Transform (FFT) spectra for the three damage modes investigated in this study, recorded from Specimen 1 during quasi-static tensile testing. The illustration is original and not reproduced from any published source. DL = delamination, MC = matrix crack, FB = fiber break events, respectively.

In this study, the main failure modes considered are Delamination (DL), Fiber Breaks (FB), and Matrix Cracks (MC), as illustrated in Figure 2. The three characteristic waveforms shown in Figure 2 are representative examples selected from Specimen 1 based on high signal-to-noise ratio and clear spectral separation, consistent with the waveform morphology criteria described in Section 3.2.1.

Delamination in CFRP composites primarily occurs through interface failure mechanisms that are triggered by mechanical loading, with fatigue and stress concentration playing critical roles; Lucas et al. [35] identified mechanical loading as a primary delamination initiator, with fatigue loading with compressive elements being more damaging than pure tensile fatigue [36]. Reza et al. [37] further clarified that delamination can occur in Mode I, Mode II and mixed-mode loading scenarios with damage features varying significantly between these conditions. Delamination events are characterized by high amplitude and exceptionally low frequency content, less than about 200 kHz, in addition to long duration, high RA and low AF. In terms of Lamb wave mode shapes, delamination events have a high ratio of antisymmetric wave mode to symmetric wave mode [8].

Fiber breaks in CFRP composites primarily occur through a complex interaction of microstructural damage mechanisms that are also triggered by mechanical loading. Fiber break events often initiate from planar fractures, thereby causing shear yield zones to propagate longitudinally during loading and changing the stress profiles for neighboring fibers, increasing their failure probability [38]. Fiber strength distribution and debonding rates are significant factors that influence the damage propagation and overall fracture toughness of the composite material [39]. Fiber break events are characterized by the highest-frequency content compared to the other failure modes, with frequencies greater than 700 kHz up to 3 MHz based on finite element simulation and correlation with actual signals obtained from experiments [8].

Matrix cracks usually originate through two competing nanoscale mechanisms, that is, fiber/matrix debonding and matrix crack initiation during mechanical loading [40]. Matrix cracks begin with pore coalescence leading to crack nucleation [41], with crack initiation triggered by local stress concentrations at fiber interfaces. In terms of frequency composition, matrix cracks have frequency contents of 250 kHz to 650 kHz. A comparison of AE frequency signatures across literature is presented in Table 1 below.

The frequency ranges cited above represent sensor-recorded frequency content rather than pure source spectra. The actual frequencies are affected by propagation effects [42], attenuation [43] and sensor frequency responses [44]. Additionally, the frequency ranges reported here should not be directly applied to different sensor types (e.g., R30 resonant transducers), thicker laminates, and different wave propagation distances.

## 3. Materials and Methods

### 3.1. Sample Preparation and Loading

Three thermoset CFRP quasi-isotropic composite rectangular coupons with layup sequence [45/90/−45/0]_2s_ from the top surface were prepared according to ASTM Standard D3039 [45]. The specimens measured 12′ × 1′ × 0.094′ with glass epoxy tabs bonded to the ends as part of a recommended practice given by the ASTM standard. The main purpose of the tabs is to prevent any damage caused by the grips of the testing machine holding the specimen.

In terms of instrumentation, AE measurements are strongly influenced by the sensor selection with clear differences observed in both the recorded waveform and corresponding frequency content. Results from previous literature showed that Piezoelectric Wafer Active Sensors (PWAS) provide superior signal-to-noise ratio at high frequencies compared to the commercially available AE transducers. PWAS can detect both symmetric and antisymmetric wave modes which are particularly useful in studies involving thin plates [46].

**Figure 3:**
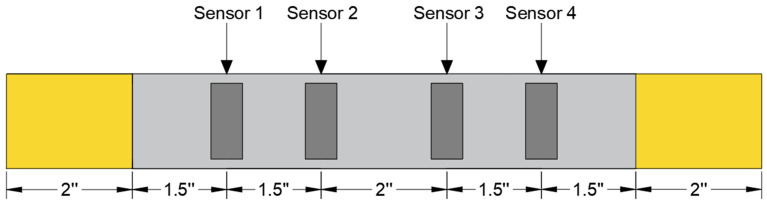
Schematic diagram of the positions of sensors.

For this study, PZT sensors are designed to maintain sensitivity over a broad range of frequencies up to 3 MHz [47]. The rectangular PZT sensors measured 15 mm × 7 mm × 0.2 mm. These sensors were adhesively bonded to the surface of the specimen using cyanoacrylate adhesive at various locations as shown below in Figure 3. Neither attenuation correction nor source-location filtering was applied during data acquisition.

Prior to testing, sensor coupling and system sensitivity were verified using Hsu–Nielsen (pencil lead break, PLB) tests [48] at multiple locations along the specimen gauge section. The PLB tests confirmed consistent signal detection across all four sensors with adequate signal-to-noise ratio (>20 dB).

The AE timing parameters were set as follows: Peak Definition Time (PDT) = 100 μs, Hit Definition Time (HDT) = 400 μs, and Hit Lockout Time (HLT) = 500 μs. These values were selected based on manufacturer recommendations for thin composite plates [12] and PLB calibration results, which showed a mean signal duration of approximately 280 μs. The selected HDT (400 μs) provides 1.4× margin above PLB duration to accommodate longer damage signals while limiting the risk of artificial merging of sequential events.

From the complete AE dataset, a total of approximately 9667 hits were recorded across 4 sensors during each test. From this total, 600 waveforms (200 per damage mode) were selected for the labeled training dataset based on the criteria described in Section 3.2.1. The selection targeted waveforms with high signal-to-noise ratio and clear waveform morphology to minimize labeling ambiguity.

The specimen was then loaded in tension monotonically in a Material Testing System (MTS) (Eden Prairie, MN, USA) system until final failure with a loading rate of 300 lbf/min. AE data from Specimen 1 were used for model development (training dataset of 600 labeled waveforms). A total of 3 specimens were tested. Specimen 2 provided the external validation dataset of 600 labeled waveforms with waveforms selected based on the same criteria used in selecting the training data. To reduce the risk of specimen overfitting, validation data (Specimen 2) were collected under nominally identical conditions (same layup, loading rate, sensor type and placement, acquisition settings) but represent an independently manufactured coupon from the same prepreg batch. While not eliminating batch level bias, this approach tests whether the model generalizes beyond the unique defect distribution (voids, resin-rich regions, fiber waviness) of the training specimen. Specimens 2 and 3 were used to generate the full-test prediction results presented later in the Results section.

**Figure 4:**
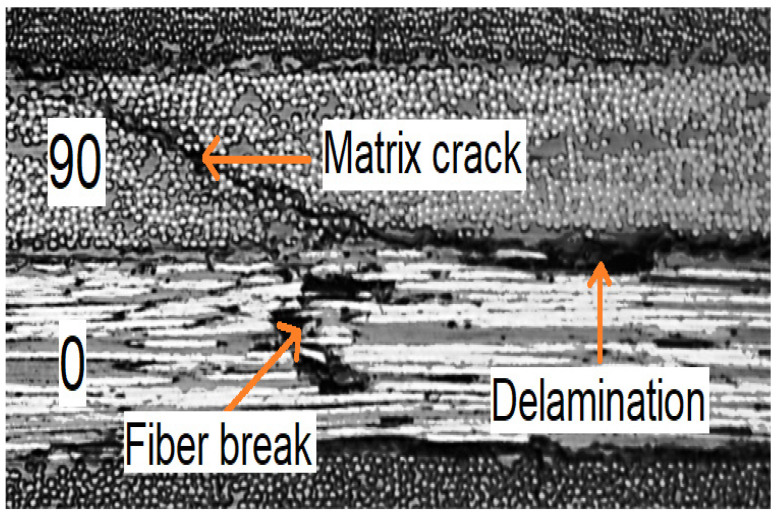
Figure showing the various damage modes in CFRP composites [8].

To accurately record the AE waveforms, the PCI-2 data acquisition system (Princeton Junction, NJ, USA) was used with PAC amplifiers. The PCI-2 system employs 18-bit analog-to-digital conversion as specified by the manufacturer. Using a threshold of 40 dB, the received signals were sampled at 20 MHz frequency and preamp gain of 60 db. A bandpass analog filter with frequency ranging from 1 kHz to 3 MHz was used to reduce noise in the received signal caused by the testing environment. The three main characteristic waveforms corresponding to matrix cracks, delamination and fiber breaks were distinguished at various load levels as illustrated below in Figure 4.

It should be noted that the AE parameters recorded by the PWAS represent the combined effects of the source mechanism, guided wave propagation through the composite plate (including dispersion, attenuation, and mode conversion), and the sensor’s own frequency response. Consequently, frequency-based parameters such as Initiation Frequency and Average Frequency reflect the sensor-convolved signal rather than the pure source spectrum. While PWAS offer broader and flatter frequency response compared to resonant transducers [46], this convolution should be considered when interpreting frequency as a direct damage discriminator.

### 3.2. Data Screening and Processing

#### 3.2.1. Reference Labeling Procedure

AE waveform labels were assigned using a multi-criteria approach combining waveform morphology analysis, load-level context, and spectral validation through Fast Fourier Transform (FFT), informed by prior finite element simulation results from previous work [8] and established literature [1,5,6,7]. Specifically, each candidate waveform was examined in the time domain for its characteristic shape: delamination events exhibit high-amplitude, long-duration signals with a dominant low-frequency carrier, fiber breaks produce short-duration, impulsive bursts, and matrix cracks generate moderate-amplitude signals with intermediate duration. The FFT of each waveform was then computed to confirm the dominant spectral content. Additionally, the load level at which the event occurred, and the spatial location (sensor channel) were used as contextual indicators, consistent with the known chronological progression of damage in quasi-isotropic CFRP laminates under monotonic tension [7,10]. It should be noted that the FFT/amplitude-based labels are representational and not equivalent to physically verified damage mechanisms.

It is acknowledged that this labeling procedure relies in part on frequency and amplitude characteristics, which are among the features subsequently evaluated by the logistic regression model. This introduces potential circularity, in that features used for labeling may appear artificially significant in the statistical analysis. To mitigate this concern, we note the following:

The labeling was performed using full waveform FFT analysis (broadband spectral content and waveform shape), whereas the logistic regression model uses only scalar parameter-based descriptors (Average Frequency, Initiation Frequency) that are simplified proxies derived from threshold-crossing counts, not direct spectral measurements. Thus, the feature representations differ between labeling and modeling.

Regarding the analysis involving Load as part of the predictors, the forward selection procedure identified Load as the single strongest predictor (Step 1, Score Chi-Square = 478.55), which was not used as a labeling criterion. This provides independent evidence that the statistical framework captures a physically meaningful structure beyond the labeling criteria.

The labels were further supported by consistency with FE simulation predictions performed by other researchers [8,49,50,51], which provide physics-based expectations for each damage mode’s spectral signature and the relative amplitude associated with it. However, these results have not been independently validated but rather correlated with actual experimental signals from these damage modes.

Nevertheless, the absence of independent physical validation (e.g., X-ray computed tomography, in situ microscopy, or digital image correlation) is a limitation of this study. Future work will incorporate such complementary validation to establish fully independent ground truth labels. The objective of statistical modeling was not to independently validate physical damage mechanisms, but to determine the minimal non-redundant subset of conventional AE descriptors sufficient to reproduce the established reference labeling under controlled test conditions.

#### 3.2.2. Feature Space Overlap and Damage Mode Classification

**Figure 5:**
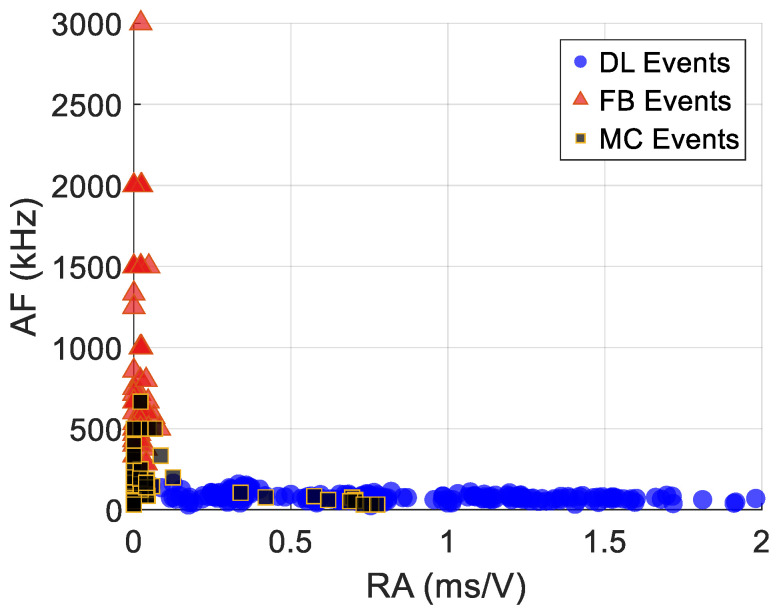
RA/AF plot for the 600 labeled training waveforms.

Figure 5 below shows the Rise Angle (RA) versus Average Frequency (AF) scatter plot for the 600 labeled training waveforms. This visualization is essential in revealing the extent of overlap between the main damage modes in the conventional AE parameter space. Some key observations are highlighted below:

Delamination (blue circles) forms a relatively distinct cluster with a high RA (>0.05 ms/V) and a low AF (<250 kHz). This reflects the slow energy release and low frequency content characteristic of interlaminar crack growth.

Fiber breaks (red triangles) concentrate at a low RA (<0.03 ms/V) and a high AF (>800 kHz), consistent with rapid brittle fracture of carbon fibers.

Matrix cracks (black squares) occupy the intermediate parameter (250–650 kHz) space with substantial overlap with both delamination events at 200–300 kHz and fiber breaks at the upper boundary at 600–700 kHz.

Visual inspection of Figure 5 reveals that approximately 20–25% of matrix crack events fall within the same AF range (500–1000 kHz) as fiber break events, creating an intrinsic classification challenge for these two damage modes as evidenced in the confusion matrices and recall values below. FFT-based dominant frequency bands used for labeling were DL with frequencies < 200 kHz, MC with frequencies of 250–650 kHz and FB events with >700 kHz. AF is a proxy and therefore its threshold needs do not match the FFT labeling bands exactly.

The visible overlap in the 500–1000 kHz AF range explains the lower classification accuracy for matrix cracks in some models compared to values for delamination and fiber breaks presented in the results section. Specifically, the logistic regression model trained shows that, approximately 19 matrix crack events were misclassified as fiber breaks, predominantly from this overlap region. This analysis demonstrates that frequency and risetime parameters alone cannot fully separate matrix crack and fiber break events, motivating the inclusion of load and amplitude as additional discriminators in the model.

### 3.3. Exploratory Studies and Label Encoding

**Figure 6:**
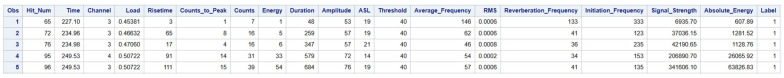
First few records of the entire dataset.

Since this study involves using only parameters, each AE waveform selected had 18 predictor variables in each column with the last column being the Label variable as shown below in Figure 6. The labels were encoded to convert the predictor “Label” from strings to integers using 1 for delamination events, 2 for fiber break events and 3 for matrix crack events. A balanced set of 600 AE waveforms were used with each failure mode having 200 observations. This analysis involved using Load as predictor.

**Figure 7:**
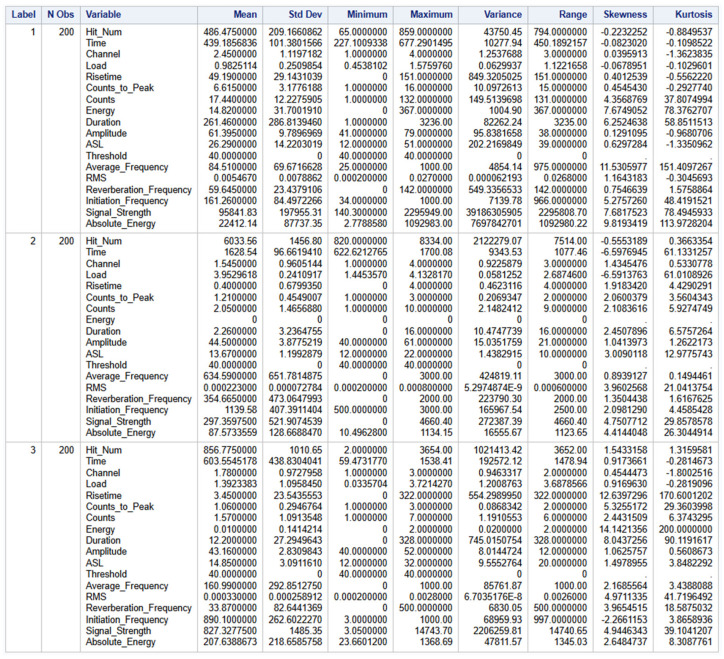
Exploratory studies of dataset.

Exploratory studies were conducted as shown in Figure 7 to understand the structure and distribution of the dataset. This was also carried out to understand the relationship between the predictors and to serve as a guide to the right analytical approach or technique to be used. The mean and standard deviation provide the central tendency and spread of the data respectively offering a quick highlight of how each variable behaves across different categories of the Label column. Maximum and minimum values help to identify the range of values in each group and to identify possible outliers or extreme data values. Skewness and Kurtosis provide insight into the shape of the distribution. Skewness gives an idea about the asymmetry of the data whilst Kurtosis measures the tailedness of the distribution that helps to assess whether the dataset follows a normal distribution or exhibits significant deviations.

### 3.4. Class Distribution in Full Dataset

The 600-waveform training set was deliberately balanced to prevent class-imbalance bias in logistic regression models. However, the actual-mode distribution in Specimen 1 based on the Gradient Boosting Model is highly imbalanced as shown below in Table 2. Training on a balanced dataset prevents the model from over-predicting the majority class (fiber break events) leading to classifications errors and model overfitting.

### 3.5. Description of Logistic Regression Model

**Figure 8:**
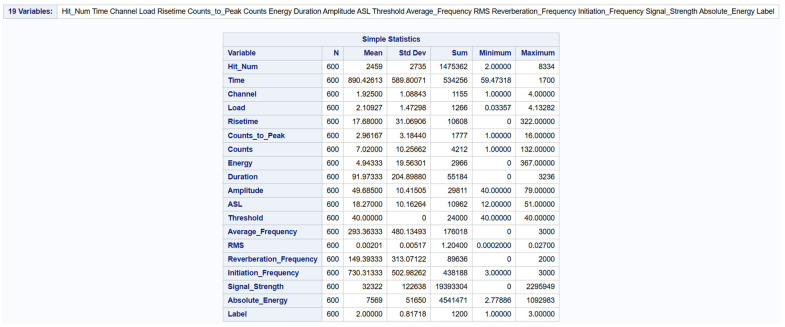
Initial number of predictors from experimental data.

Inferential statistics is a statistical method that involves making predictions and conclusions about a population based on sample data. With this, each member of the population has an equal chance of being used. A logistic regression model was developed to make predictions based on the independent variables given. The model started with a total of 19 variables as illustrated in Figure 8.

**Figure 9:**
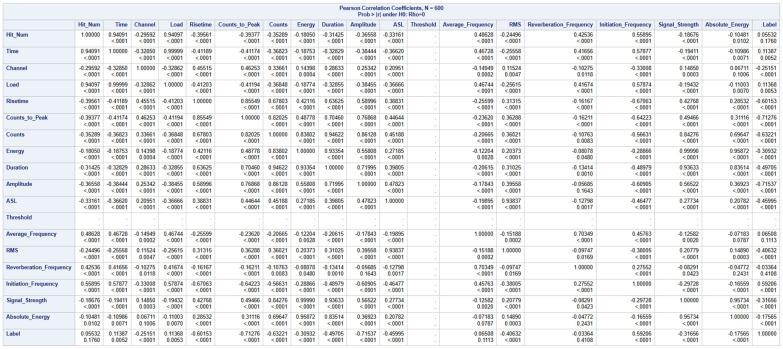
Pearson Correlation Coefficient Summary.

To begin the process, a correlation process was run to identify the levels of correlations between the predictor variables among themselves as shown in Figure 9.

To address the issue of multicollinearity among the predictors, the correlation matrix was well examined. Any pair of variables with an absolute correlation coefficient that exceeded 0.8 [52] was flagged. The threshold of |r| > 0.8 for identifying highly correlated predictor pairs was adopted following established guidelines for multicollinearity screening. While alternative thresholds (e.g., 0.7 or 0.9) or variance inflation factor (VIF) analysis could be employed, the sensitivity of the results to this choice is expected to be limited because the forward selection procedure with Type 3 analysis provides an additional layer of redundancy detection that is independent of the correlation threshold.

**Figure 10:**
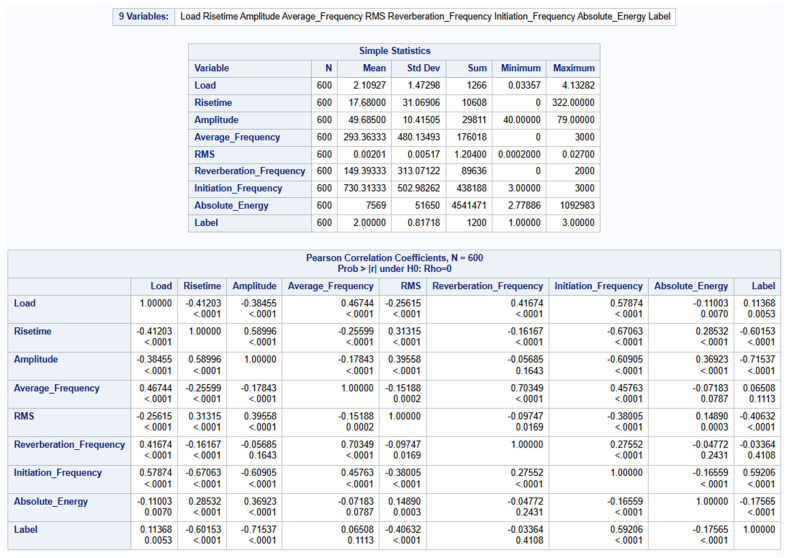
Reduced number of variables to be used for the logistic regression model.

From each of the highly correlated pair, one variable was removed leading to the removal of the following variables: ‘Hit_Num’, ‘Duration’, ‘Counts_to_Peak’, ‘Signal_Strength’, ‘Time’, ‘Energy’, ‘Counts’ and ‘ASL’. The threshold predictor was also dropped because it had a constant value of 40 in the dataset irrespective of the ‘Label’ value. This statistical technique reduced the number of variables to nine variables as shown in Figure 10.

A new dataset was created based on these nine variables to create the logistic regression model. A multinomial logistic regression model was used with a reference category of 2 indicating fiber break failure mode. This mode was chosen because it is the most critical of all the failure modes. That is, when clusters of fiber breaks are recorded, it indicates that the specimen is nearing its final failure. The forward selection technique was used, which involves beginning with no predictor variables, just the intercept. Predictor variables are then added based on a selection criterion such as *p*-value, AIC or BIC and reevaluated after the addition. Additional variables are added and tested only if they significantly improve the model. This process continues until there are no more variables that when added improve the model.

## 4. Results and Discussion

The optimization technique used in the model is the Newton–Raphson technique. This is an iterative technique used to estimate the maximum likelihood estimates of the model parameters. It begins with an initial estimate of the parameters and then updates them by computing the gradient of the log likelihood function indicating the direction of improvement and the second derivative which adjusts the step size. This process repeats until the estimates become stable.

At step 0, the Gradient Convergence Criterion (GCONV) converges; −2 Log L (−2 log likelihood) is a statistic in logistic regression that measures how well the model fits the given data. The lower the value of −2 Log L, the better the model fits the data. Residuals are defined as the difference between the actual values of the dependent variable (Label) and the values predicted by the regression model. The Residual Chi-Square Test is used to assess whether the predictor variables in the model significantly improve the model fit compared with the model with only the intercept. The calculated *p*-value indicates the significance of the test. A very small *p*-value (<0.05) shows that predictor variables improve the model significantly.

### 4.1. Forward Selection and Model Convergence Process

The Akaike Information Criterion (AIC) is a technique used for model selection. A lower value indicates a better fit of the model. Comparing the values of AIC, that of the intercept alone had a value of 1322.335. When the covariate was added, it decreased to 665.399, indicating a significant improvement in the fitness of the model. The Schwarz Criterion (SC) is a similar technique to AIC but includes a penalty for the number of parameters. A lower value also indicates a better model fit. As shown below, the value of SC for with the covariates added is better than the model with just the intercept only. A similar pattern is also seen for the −2 Log L criteria. Regarding testing the global null hypothesis, the Likelihood Ratio Test compares the likelihood of the full model with the intercept-only model. A Chi-Square value of 660.9355, *p* < 0.0001, indicates strong evidence that the predictor variable added is significant with regard to the Label outcome. The score test is used to check whether any predictor variable significantly improves the model fit. A Chi-Square value of 478.5455, *p* < 0.0001, suggests that the predictor variables are significant. The Wald test evaluates the significance of coefficients individually. A Chi-Square value of 51.2324, *p* < 0.001, also indicates the significance of the predictor variables. The Residual Chi-Square Test tests whether the model adequately fits the data. A Chi-Square value of 334.5888, *p* < 0.0001, suggests that there are indeed other predictors not yet in the model that are significantly associated with the Label variable.

**Figure 11:**
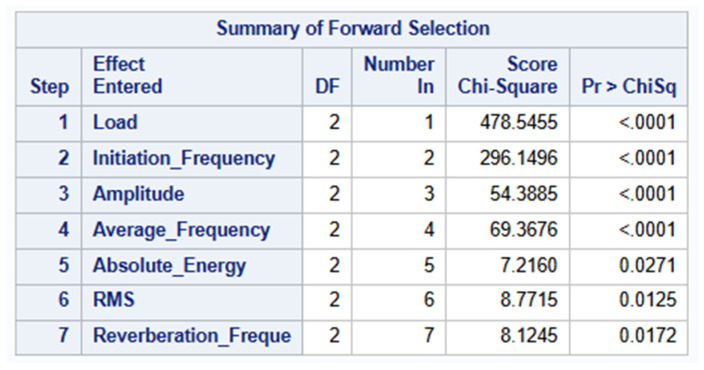
Summary of Forward Selection Process.

This forward process continues for all the predictor variables. For Steps 5, 6 and 7, the model starts adding weaker but still statistically significant predictors. The values of the Chi-Square are much smaller and their *p*-values still below the significance threshold of 0.05, making them still statistically significant. However, their contribution to the predictive power of the model is relatively smaller than the first four steps involving the predictors: Load, Initiation Frequency, Amplitude and Average Frequency. The forward selection process stops after the seventh step because there are no additional variables that, when added to the model, meet the significance threshold of 0.05 required for entry into the model. In summary, the AIC values decrease significantly from 665.399 to 182.039, indicating each added predictor variable significantly improves the model. In addition, −2 Log L decreases from 657.399 to 150.039, further confirming better model fit. The Wald tests, Score and Likelihood Ratio are significant (<0.05), indicating that the predictor variables contribute significantly to the model. Figure 11 below summarizes the forward selection process.

#### Quasi-Complete Separation

Beginning at Step 5, the SAS Studio Release 3.82 (Enterprise Edition) output reports “Quasi-complete separation of data points detected.” This condition arises when a linear combination of predictors nearly perfectly separates one or more response categories, causing the maximum likelihood estimates to diverge toward infinity for the separating coefficients. Consequences include inflated standard errors, potentially unreliable coefficient estimates, and misleading *p*-values for the affected parameters [53,54].

Several observations mitigate the impact of this issue on our conclusions. First, quasi-complete separation was detected only after the four core predictors (Load, Initiation Frequency, Amplitude, Average Frequency) had already entered the model at Steps 1–4 with proper convergence (GCONV = 1E-8 satisfied). The separation arises from the addition of Absolute Energy, RMS, and Reverberation Frequency, precisely the predictors that the subsequent Type 3 analysis identified as non-significant (*p* > 0.05). Thus, the separation reinforces rather than contradicts the conclusion that the model should be simplified to four predictors.

Secondly, to verify the robustness of the four-predictor model, we confirmed that the reduced model (containing only Load, Initiation Frequency, Amplitude, and Average Frequency) achieves proper convergence without separation warnings, producing stable coefficient estimates. This is seen in the additional figures below with the rows corresponding to these four predictors.

As an additional safeguard, Firth’s penalized likelihood estimation [54,55] could be applied to obtain bias-corrected estimates. This is recommended for future studies employing the full seven-predictor model but is not required for the simplified four-predictor model advocated here.

### 4.2. Type 3 Analysis

**Figure 12:**
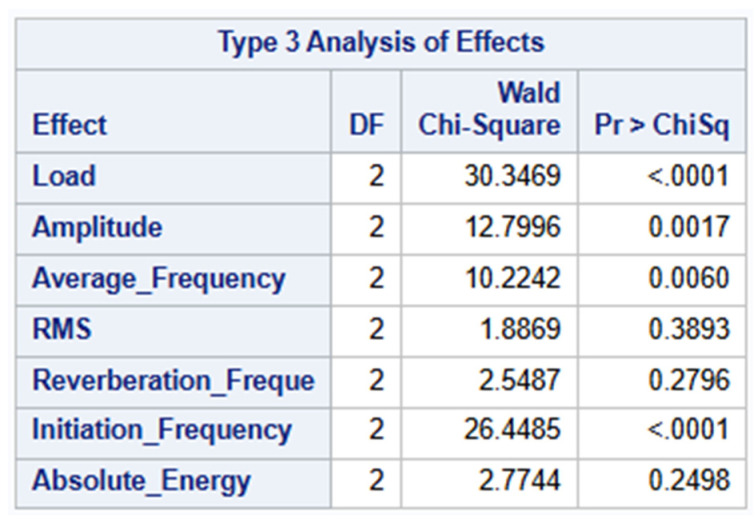
Type 3 Analysis of Effects Before Simplification of Model.

Figure 12 below summarizes the Type 3 Analysis of Effects. This evaluates the significance of each predictor variable while controlling for all the other variables in the model. It is used to determine how significantly each predictor variable contributes to the model. The stepwise forward selection from the earlier process identified seven variables as significant when added individually, with Load and Initiation Frequency being the strongest initial predictors. However, in Type 3 analysis, it is revealed that only Load, Initiation Frequency, Amplitude and Average Frequency provide unique and statistically significant contributions to the model. The other remaining variables are not significant in the full model indicating that their predictive power is redundant. This suggests that the final model might be overfitting and could be simplified by retaining only the four key significant predictors. Further simplification of the model by removing the non-significant predictors such as RMS, Reverberation Frequency and Absolute Energy was carried out.

The statistical significance of the frequency-based parameters (Initiation Frequency, Average Frequency) must be interpreted under the condition that these represent sensor-measured content rather than source spectra. The observed frequency ranges reflect

- Source physics (damage area);
- Propagation effects such as dispersion and attenuation;
- PWAS sensor response;
- Analog source filtering.

Consequently, the four-parameter model is sensor-specific and requires recalibration as the sensor type, sensor position or acquisition settings change.

### 4.3. Maximum Likelihood Estimates

**Figure 13:**
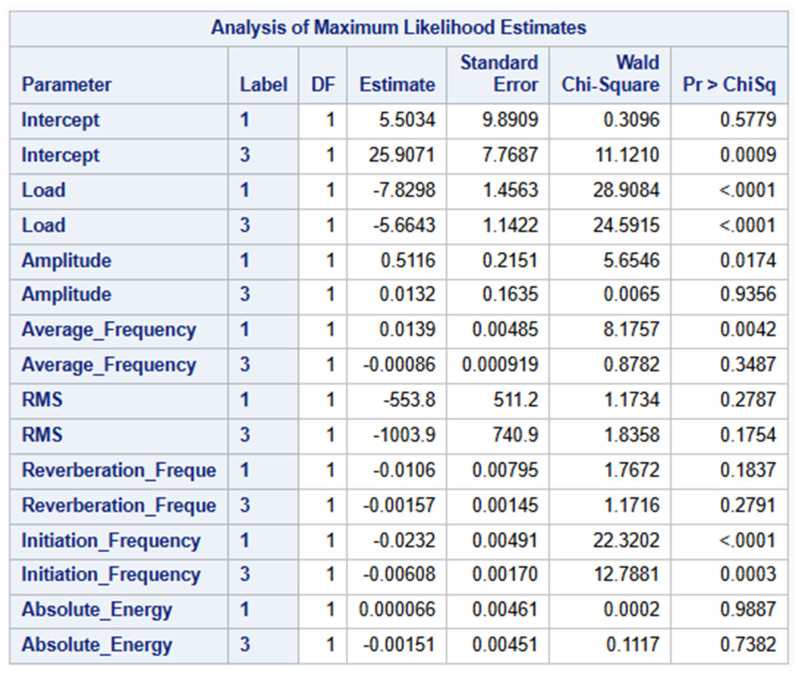
Analysis of Maximum Likelihood Estimates.

The Analysis of Maximum Likelihood estimates confirm the results from the Type 3 analysis that reveal the specific levels of the categorical predictors that are driving the model’s predictions. The highly significant predictors such as Load and Initiation Frequency show strong, significant effects for both of their parameter estimates, thus confirming their consistent strength across the outcome categories. In contrast, the weak predictors showed inconsistent results for the outcome categories. For example, Amplitude and Average Frequency are significant for only one of the two outcome categories. The non-significant predictors from the Type 3 analysis above show consistently that they contribute no meaningful and unique significance to the overall model. Figure 13 below summarizes the parameters and values involved in the Analysis of Maximum Likelihood.

**Figure 14:**
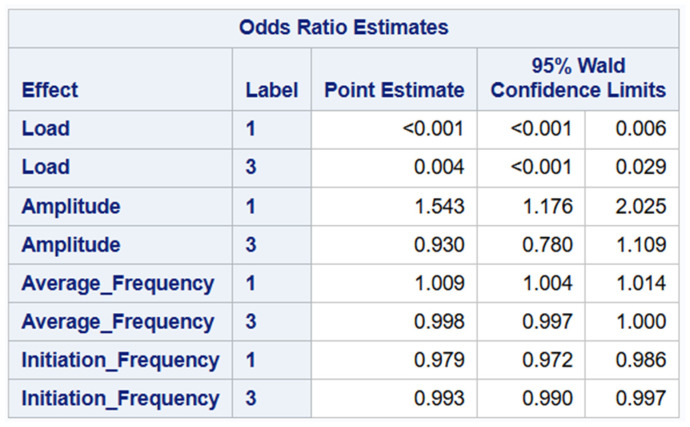
Odds ratio estimates for the reduced four-predictor model.

In the reduced four-predictor logistic regression model, the odds ratios (ORs) with 95% Wald confidence intervals (CIs) indicate predictor-specific effects for Label 1 (delamination events) and Label 3 (matrix crack events). Load was associated with significantly lower odds of both Label 1 (OR < 0.001, 95% CI: <0.001–0.006) and Label 3 (OR = 0.004, 95% CI: <0.001–0.029) relative to Label 2. Amplitude significantly increased the odds of Label 1 (OR = 1.543, 95% CI: 1.176–2.025) but showed no significant association with Label 3 (OR = 0.930, 95% CI: 0.780–1.109). Average Frequency significantly elevated the odds of Label 1 (OR = 1.009, 95% CI: 1.004–1.014), whereas it had no meaningful effect on Label 3 (OR = 0.998, 95% CI: 0.997–1.000). Initiation Frequency significantly reduced the odds of both Label 1 (OR = 0.979, 95% CI: 0.972–0.986) and Label 3 (OR = 0.993, 95% CI: 0.990–0.997), indicating a consistent protective effect across both non-reference outcome categories as shown below in Figure 14.

### 4.4. Confusion Matrix

**Figure 15:**
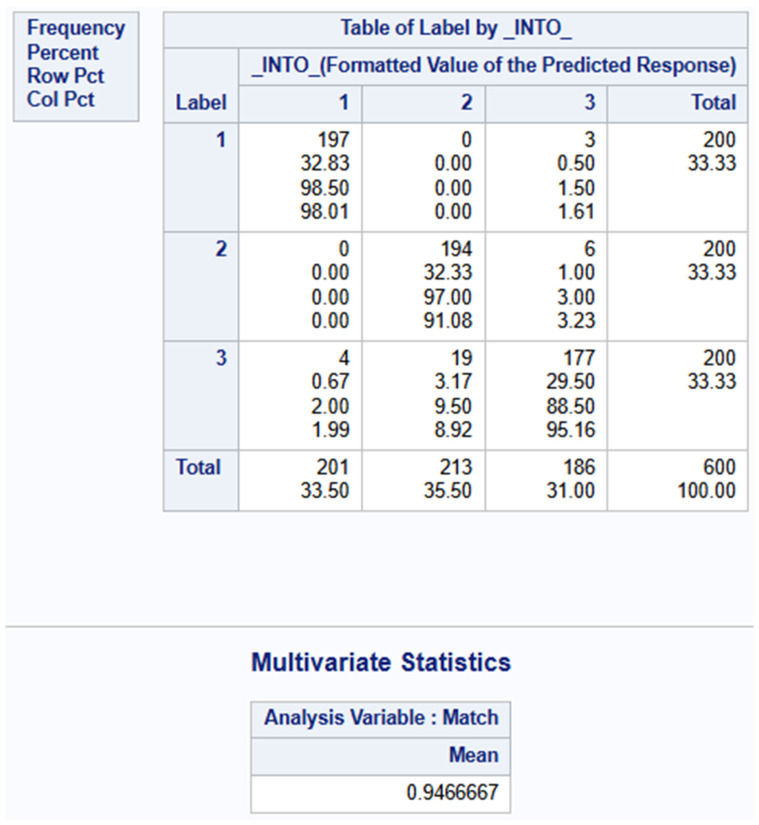
Confusion Matrix for Multivariate Regression Model.

The model demonstrates excellent overall predictive accuracy of 94.67%, as shown in the classification table in Figure 15. However, this performance is unevenly distributed across outcome categories. The model performs exceptionally well in identifying Label 1 (delamination events) with 98.5% accuracy and Label 2 (fiber break events) with 97.0% accuracy but shows more difficulty with Label 3 (matrix crack events), with an accuracy of 88.5%. For Label 1, recall was 98.50% (197/200) with precision of 98.01% (197/201). Label 2 showed a recall of 97.00% (194/200) and precision of 91.08% (194/213). Label 3 demonstrated a recall of 88.50% (177/200) and precision of 95.16% (177/186). Overall accuracy was 94.67%, with a macro-average F1-score of approximately 94.64%.

The primary confusion occurs between adjacent categories, particularly in misclassifying actual Label 3 observations as Label 2, suggesting the model finds these categories more challenging to distinguish. This situation is expected due to the similarities in relative amplitude and frequency between matrix crack events and fiber break events While the high overall accuracy indicates a strong model, the pattern of misclassification does not significantly affect the overall model performance.

### 4.5. Model Implementation

#### 4.5.1. Machine Learning (ML) Algorithms

To test the statistically significant predictors obtained from the logistic regression model in SAS, the same predictors were used to build and test different ML models. The models were Logistic Regression, Random Forest, Gradient Boosting, Support Vector Machine, k-Nearest Neighbors and Naïve Bayes. Each model’s parameters were obtained based on optimum hyperparameter tuning to obtain the best results possible. The main aim of these models was to be able to correctly predict the damage mechanism occurring based on the conventional AE predictors. The dataset used consists of 200 cases each of the damage mechanisms. The dataset was split into 480 samples for training and 120 samples for testing. In the training for all the models, 5-fold cross-validation was used to further refine the models.

Prior to training the ML and DL models, all input features were standardized to zero mean and unit variance using scikit-learn’s StandardScaler, fitted on the training partition only to prevent data leakage. Standardization is essential for distance-based algorithms (SVM, kNN) and gradient-based optimization (neural networks) and ensures comparable coefficient scales in logistic regression. For tree-based models (Random Forest, Gradient Boosting), standardization does not affect performance but was applied uniformly for consistency across all models. For hyperparameter tuning, the GridSearchCV from scikit-learn was used together with stratified 5-fold cross-validation. Random seed of 42 was used for all models for reproducibility with StandardScaler fitted on training folds only during cross-validation and applied to the validation fold to prevent data leakage.

**Figure 16:**
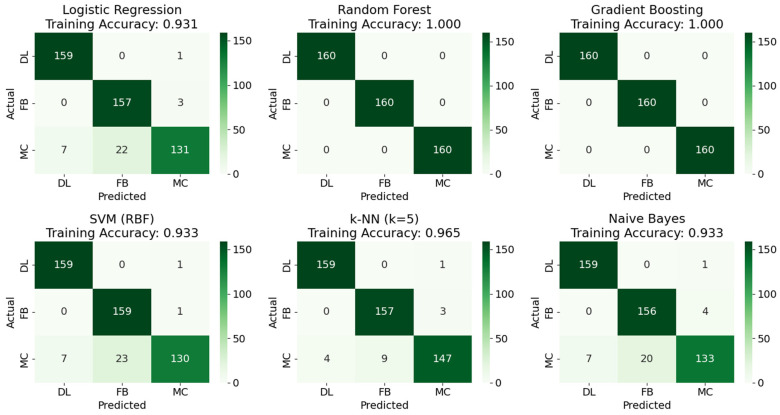
Confusion matrices for ML algorithms based on training data.

**Figure 17:**
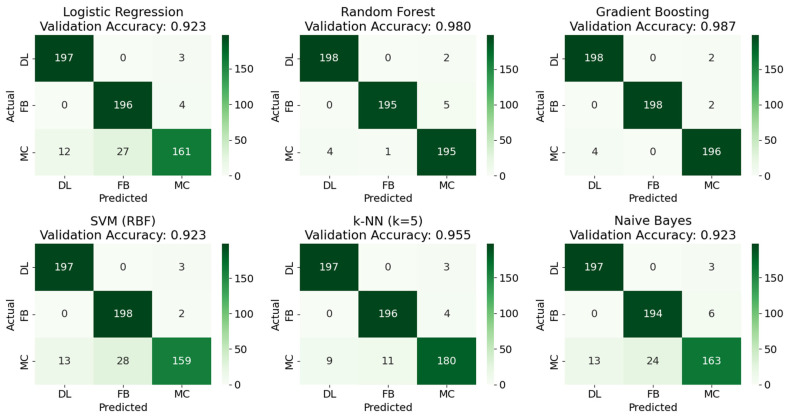
Confusion matrices for ML algorithms based on external validation data.

Figure 16 below shows the confusion matrices for the various models based on the training data. The labels FB signified fiber breaks, DL and MC for delamination and matrix crack, respectively. Some models such as Random Forest and Gradient Boosting showed 100% accuracy, followed by kNN with 96.5% accuracy and the rest of the models with an average accuracy of about 93.2%. The trained models were then validated using 600 samples of external data that none of the models had seen, as shown below in Figure 17. The validation data source was from another specimen with a similar layup and labeling procedure to ensure that the models were not overfitting to just one type of specimen. The external validation merely confirms the models’ stability across similar layups under identical testing and labeling conditions. Similar to the results from training, Gradient Boosting and Random Forest had the highest accuracy of 98.7% and 98%, respectively, followed by the rest of the models. To provide a more comprehensive evaluation beyond overall accuracy, Table 3 reports the per-class precision, recall, and F1-score for each model. These metrics reveal that the systematic misclassification of matrix crack events as fiber breaks, observed across multiple models, reduces MC recall to approximately 81% for Naïve Bayes and Logistic Regression Models, while DL and FB classes consistently achieve recall above 97% in all the models. This could be due to linear decision boundaries struggling to separate the MC/FB overlap zone as seen in the RA-AF plot in Figure 5. It also reflects genuine physical overlap in the feature space, which the nonlinear boundaries of tree-based models were better able to partition.

#### 4.5.2. Deep Learning (DL) Algorithms

Four DL models were created to test the statistically significant predictor’s ability to correctly predict damage mechanisms. The models evaluated are fully connected feedforward neural networks motivated by the low-dimensional and non-spatial nature of the input data [56]. Fully connected networks impose minimal inductive bias while retaining expressive capacity to model nonlinear interactions among predictors. Other complex architectures such as Convolutional Networks and Recurrent Networks were not considered as their structural assumptions are not aligned with the characteristics of the feature space and hence introduced unnecessary complexity [57]. Therefore, multilayer perceptrons provide an appropriate balance between representational power, computational efficiency and generalization performance for the multi-class classification task considered [58]. Table 4 below summarizes the model architecture and key characteristics of the various models used.

**Figure 18:**
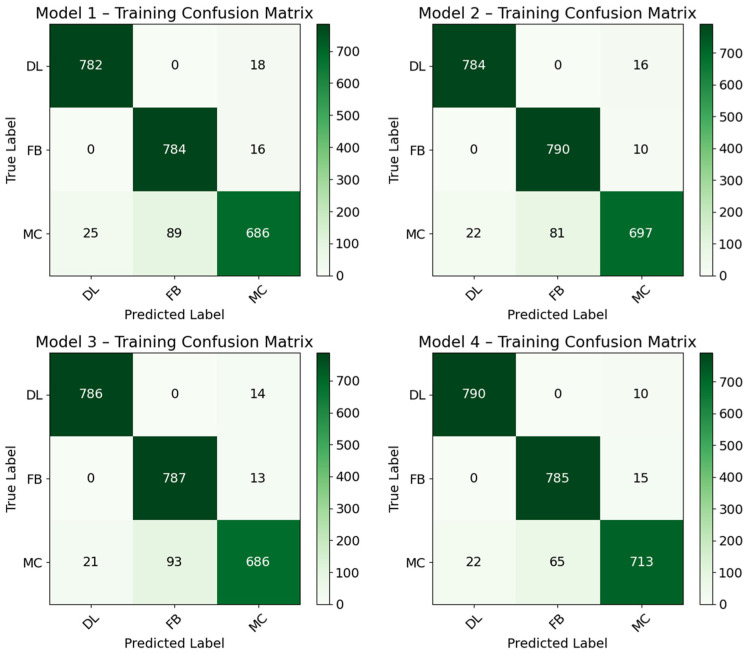
Confusion matrices for DL models based on training and testing data.

**Figure 19:**
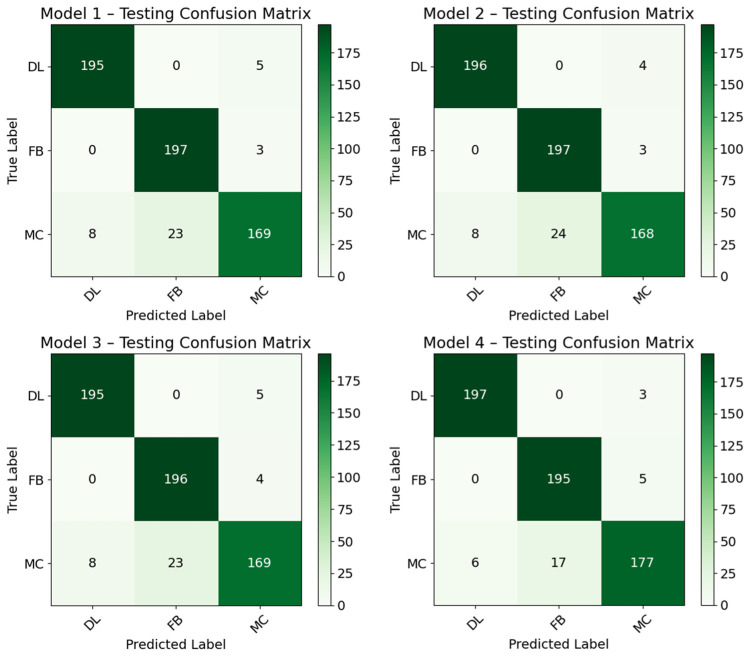
Confusion matrices for DL models based on validation data.

**Figure 20:**
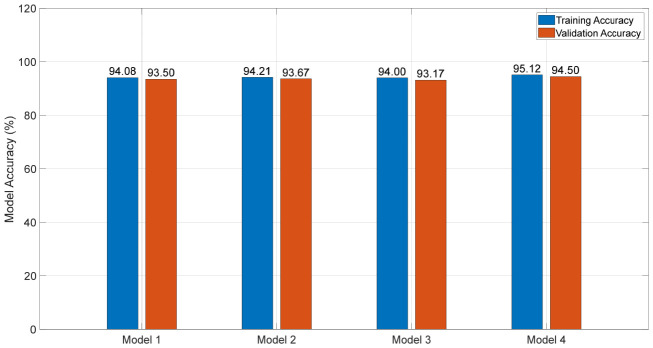
Training and Validation Accuracies for DL Models.

For the DL models, 5-fold cross-validation was used. This means that initially, the entire dataset of 600 samples was divided into five equal parts. Each fold uses 4/5 of the data for training which is 4/5 × 600 leading to 480 samples. Since there are 5 folds, each fold’s training confusion matrix is calculated based on its 480 training samples, making the sum of all the training confusion matrices 5 × 480, making 2400 samples in total, as illustrated in Figure 18 below. The sum (2400) represents the total number of training predictions that were made across all the folds, not the total number of unique samples, that is, 600, that the models began with. The DL models were further validated with external data as shown by the confusion matrices in Figure 19. The accuracies for training and validation are compared below in Figure 20 with the least-performing model having an accuracy of 93.17%.

It is acknowledged that, for a classification task with only four input features and 600 training samples, deep learning models carry a higher risk of overfitting compared to simpler ML approaches. Indeed, the DL models (validation accuracy 93.2–94.5%) did not outperform the best ML models (Gradient Boosting: 98.7%, Random Forest: 98.0%), consistent with recent findings that tree-based ensemble methods often match or exceed neural networks on low-dimensional tabular data [57]. The inclusion of DL models in this study serves primarily to demonstrate that the four-predictor feature set is sufficient across diverse classifier architectures, rather than to advocate for deep learning as the preferred approach for this specific task. For practical SHM deployment with the identified feature set, simpler models such as Gradient Boosting or Random Forest are recommended given their superior accuracy, interpretability, and computational efficiency.

### 4.6. Significance in Damage Evolution

Following the accuracies of the deep learning and machine learning models, entire test data was fed into these models to predict the failure modes during the test. The results from the classification are summarized in Table 5 below. The table presents an important illustration for damage progression. These plots should be interpreted with the caveat that they represent the model’s classification of AE events according to the reference labeling scheme not independent validation. It is observed that, in both specimens, the Cumulative Hits for fiber breaks increase exponentially towards the failure load. In addition, a similar trend is seen in the amplitude plots where there is an increased amount of fiber breaks towards the end. This confirms results in the previous literature [10] where an increase in fiber break events gives an indication of the imminent failure of the specimen.

### 4.7. Role of Load as a Predictor

It is important to note that Load is not an AE signal descriptor but rather an external mechanical variable recorded synchronously with AE data. Its inclusion in the predictor set is motivated by the fact that commercial AE systems routinely record parametric inputs (load, displacement, temperature) alongside signal descriptors, and in practice, these are available during monitoring of proof tests and qualification loading.

The finding that Load enters first in the forward selection (Chi-Square Score = 478.55) is physically expected. Different damage mechanisms are activated preferentially at different load levels, with matrix cracking initiating at lower loads and fiber breaks concentrating near ultimate failure [1,7,10]. While this result is not novel in itself, it provides quantitative confirmation of the load dependence of damage mode activation and validates the statistical framework’s ability to recover known physical relationships.

However, in real-world SHM deployments, precise real-time load information may not always be available, for example, under complex multi-axial loading, fatigue, or thermal–mechanical coupling. These types of loading, amongst several factors, limit direct transfer to operational SHM systems. To assess the model’s generalizability in scenarios without Load, the forward selection analysis was repeated, excluding Load from the candidate predictor set, and then retraining the ML and DL models using the new set of predictors. The comparison of the results with and without the load predictor is summarized below in Table 6.

While training was performed on a balanced dataset for classifier stability, practical deployment would require consideration of the naturally imbalanced damage distribution observed in full-test data. Table 7 presents per-class metrics for the no-load condition, revealing critical insights into model behavior and the discriminative role of the Load parameter. The most visible pattern is the severe reduction in fiber break recall for linear models when Load is removed. For example, FB recall for Logistic Regression drops from 98% with load to 52% without load. In addition, FB recall drops from 99% to 50% and 97% to 49% for SVM and Naïve Bayes Models with and without Load, respectively. Simultaneously, the MC recall values increased overall from the Load case to the No-Load case. This inverse pattern indicates systematic MC and FB misclassification. Without the load information, linear models cannot separate the MC/FB overlap zone and defaults to classify ambiguous events as matrix crack events. In contrast, the ensemble models maintain consistently high performance for the No-Load case. Additionally, delamination classification remains robust across all models, confirming delamination’s unique signature (high amplitude with low frequency). This provides sufficient discriminability without any temporal context.

## 5. Conclusions

The model-building process, using forward selection, identified a set of seven candidate predictor variables that collectively and significantly explain the model. The present analysis evaluates the statistical sufficiency of conventional AE parameters within the adopted reference labeling framework, rather than independently verifying physical damage mechanisms. The hierarchy of variable importance was clearly established, with Load and Initiation Frequency emerging as the dominant predictors, followed by Amplitude and Average Frequency and the rest of the predictors for the analysis involving Load as a predictor. However, the final Type 3 analysis revealed a critical insight; that is, when all variables are considered together, only these first four provide unique, statistically significant information. The remaining three variables, Absolute Energy, RMS, and Reverberation Frequency, become statistically non-significant in the full model, indicating that their apparent individual significance was due to overlapping information with the core predictors. This suggests that while the forward selection process was effective at identifying candidate variables, the final model can be simplified without losing predictive power. The identified parameter importance is conditional on the measurement configuration employed; that is, different sensors (for example, R30 resonant sensors) will change the recorded frequency content. This may potentially change the relative importance of Initiation Frequency compared to amplitude. The four-parameter set should be considered a starting point for sensor-specific optimization rather than a universal solution.

The results from the ML and DL models provide evidence that the labeled AE waveform classes can be effectively classified using only four conventional AE parameters with validation accuracies up to 98.7%. It is also important to note that the 94.67% accuracy obtained by the logistic regression model assumes synchronous load recording. In scenarios where load is unavailable, the accuracy drops to 84.76%. In cases of SHM deployment with the absence of synchronous load recording, Gradient Boosting and Random Forest give the highest validation accuracy of 98% with the alternative four-parameter set, that is, Amplitude, Reverberation Frequency, Initiation Frequency and Absolute Energy.

Interpreting it in terms of damage characterization in CFRP composites, Load level, frequency and amplitude are the key features for classification. This is a confirmation of the previous literature on the increase in fiber break events as the load increases [10] in addition to high-frequency components. Delamination events had high amplitude and low frequency content compared to matrix crack events. These findings provide quantitative validation for commonly employed AE selection parameters fed as input into various algorithms and models. Limiting the model to a small number of dominant predictors reduces the computational costs involved, mitigating overfitting and enhancing robustness under varying operation conditions.

A key limitation of this study is the absence of independent physical validation of ground truth labels. Postmortem microscopy and in situ imaging techniques such as digital image correlation (DIC) or X-ray CT were not employed. Incorporating such methods in future studies would provide fully independent label verification and eliminate potential circularity between labeling criteria and feature selection outcomes. Another limitation of this study is the small specimen count (*n* = 3), which poses risks to statistical generalizability and model robustness. While 600 labeled waveforms per specimen provided sufficient specimen statistical power for logistic regression, the model was trained on data from a single specimen and validated on only one external specimen with the same layup and loading rate. In addition, there is a significant decrease in accuracy when comparing the cases with synchronous load to those without.

Future work will focus on the limitations of this study by validating the robustness of the proposed features across different laminate configurations, different load levels, and, in addition, extending the study to multi-class damage classification and progressive damage tracking. Larger-scale studies with multiple specimens, material systems, and independent labeling validation are needed before deployment in operational SHM systems. These efforts will further clarify the generalizability of the identified predictors, supporting the development of reliable and physics-informed AE-based diagnostics for advanced composite materials.

## Figures and Tables

**Table 1:** Comparison of AE Frequency Signatures Across Literature.

Study	Material	Sensor Type	DL Frequency (kHz)	MC Frequency (kHz)	FB Frequency (kHz)	Notes
Peter de Groot et al. [5]	T300/914 carbon/epoxy unidirectional and cross ply	Broadband sensor PAC model WD	>280	150–450	350–550	Used real-time FFT
Mizutani et al. [6]	T8—H/3900 carbon/epoxy cross ply	PICO sensor	100–300	100–400	300–700	Considerable overlap exists between damage modes
Liu et al. [7]	T700/8911	R15 I resonant sensor (150 kHz)	RA-AF clustering used (no frequency bands)			Sensor bandwidth limited
Rijal et al. [8]	AS4/3501-6 simulation	Ideal point receiver	<250	250–650	>700	Pure source spectrum
This study	Quasi-isotropic carbon epoxy	PWAS	<200	250–650	>700	Sensor convolved, includes dispersion

**Table 2:** Predicted Class Distribution in Full Specimen 1 Dataset.

Class	Count	Percentage
Matrix Cracks (MC)	323	3.34%
Fiber Break (FB)	7332	75.86%
Delamination (DL)	491	5.08%
Unclassified Events, Mixed Events and Noise	1521	15.73%

**Table 3:** Per-Class Classification Metrics for ML Models based on Validation Data.

Model	Class	Precision	Recall	F1-Score
Logistic Regression	DL	0.94	0.98	0.96
	FB	0.88	0.98	0.93
	MC	0.96	0.81	0.88
Random Forest	DL	0.98	0.99	0.99
	FB	0.99	0.97	0.98
	MC	0.97	0.97	0.97
Gradient Boosting	DL	0.98	0.99	0.99
	FB	1.0	0.99	0.99
	MC	0.98	0.98	0.98
SVM	DL	0.94	0.98	0.96
	FB	0.88	0.99	0.93
	MC	0.97	0.80	0.87
k-NN (k = 5)	DL	0.96	0.98	0.97
	FB	0.95	0.98	0.96
	MC	0.96	0.90	0.93
Naïve Bayes	DL	0.94	0.98	0.96
	FB	0.89	0.97	0.93
	MC	0.95	0.81	0.88

**Table 4:** Table Summarizing DL Models’ Architecture and Key Characteristics.

Model No.	Model Architecture	Model Characteristics
1	Input (4) Dense (32, ReLU) Dense (16, ReLU) Dense (3, Softmax)	Low risk of overfitting Fast convergence Serves as baseline
2	Input (4) Dense (64, ReLU) Dropout (0.3) Dense (32, ReLU) Dropout (0.3) Dense (16, ReLU) Dense (3, Softmax)	Higher capacity Explicit regularization Improved generalization on nonlinear boundaries
3	Input (4) Dense (64, BatchNorm, ReLU) Dense (32, BatchNorm, ReLU) Dense (3, Softmax)	Reduced internal covariate shift. More stable optimization Effective at moderate depth
4	Input (4) Dense (128, ReLU) Dense (64, ReLU) Dense (32, ReLU) Dense (3, Softmax)	High expressiveness Best suited for complex class boundaries Benefits from balanced data

**Table 5:** Summary of Cumulative Hits and Amplitude Plots.

Specimen Number	Cumulative Hits	Amplitude Versus Load
2	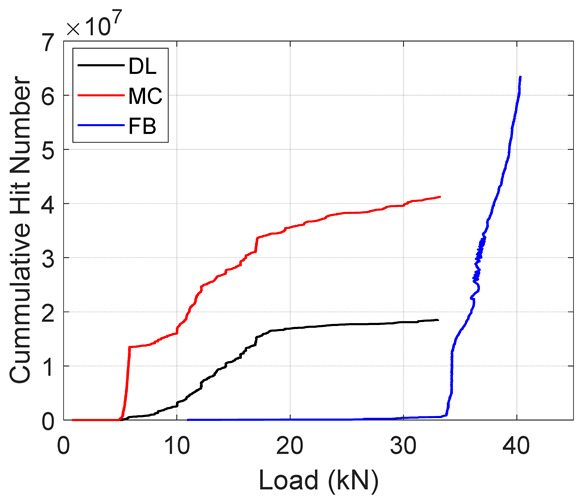	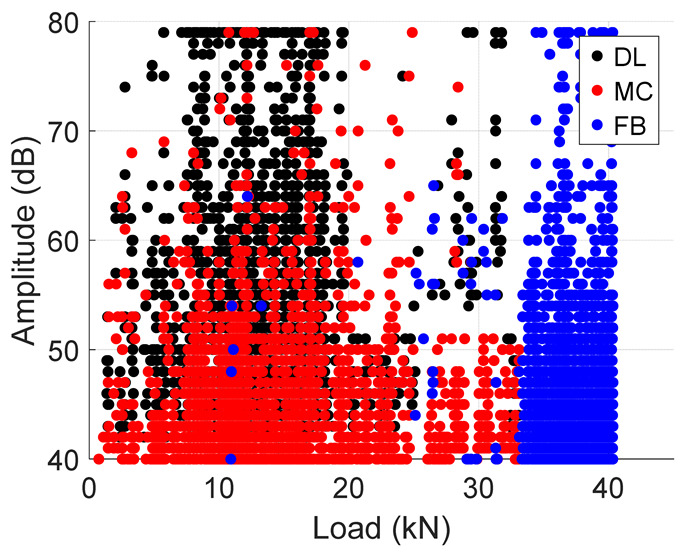
3	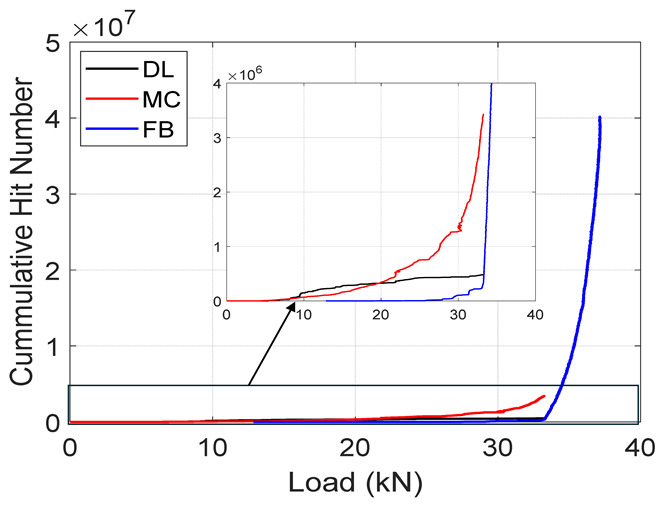	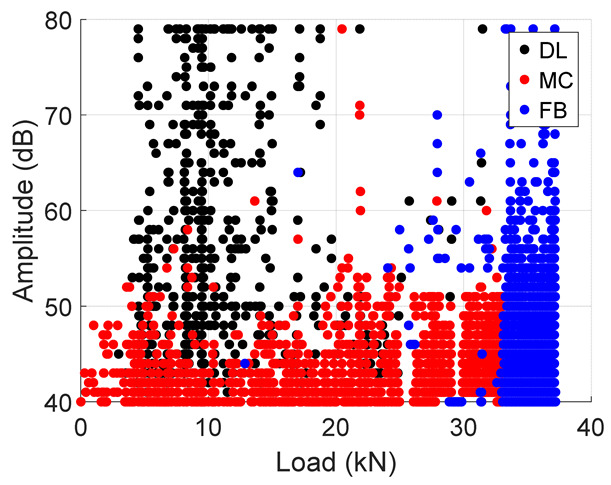

**Table 6:** Comparison of models with and without the load predictor.

Parameter	With Load Predictor	Without Load Predictor
Highly Significant Predictors	Load	Amplitude
	Amplitude	Reverberation Frequency
	Average Frequency	Initiation Frequency
	Initiation Frequency	Absolute Energy
Logistic Regression Model Accuracy	94.67%	84.76%
Machine Learning Models		
Logistic Regression	0.923	0.795
Random Forest	0.980	0.980
Gradient Boosting	0.987	0.980
SVM	0.923	0.797
k-NN (k = 5)	0.955	0.935
Naïve Bayes	0.923	0.785
Deep Learning Models		
Model 1	0.935	0.8017
Model 2	0.9367	0.8050
Model 3	0.9317	0.8017
Model 4	0.945	0.8033

**Table 7:** Per-Class Classification Metrics for ML Models based on the No-Load Condition.

Model	Class	Precision	Recall	F1-Score
Logistic Regression	DL	0.94	0.98	0.96
	FB	0.90	0.52	0.66
	MC	0.64	0.89	0.74
Random Forest	DL	0.99	0.99	0.99
	FB	0.99	0.97	0.98
	MC	0.97	0.97	0.97
Gradient Boosting	DL	0.99	0.99	0.99
	FB	0.99	0.96	0.98
	MC	0.96	0.98	0.97
SVM	DL	0.94	0.97	0.96
	FB	0.95	0.50	0.66
	MC	0.64	0.92	0.75
k-NN (k = 5)	DL	0.96	0.98	0.97
	FB	0.94	0.94	0.94
	MC	0.91	0.89	0.90
Naïve Bayes	DL	0.98	0.94	0.96
	FB	0.88	0.49	0.63
	MC	0.62	0.93	0.74

## Data Availability

The original contributions presented in this study are included in the article. Further inquiries can be directed to the corresponding author.

## References

[1] Saeedifar M., Zarouchas D. (2020). Damage characterization of laminated composites using acoustic emission: A review. Compos. Part B.

[2] Dhar P., Rijal M., Amevorku R.D., Amoateng-Mensah D. (2025). Assessing Impact Damage In Thermoplastic Composites Using Maximum Contrast Approach. IOSR J. Mech. Civ. Eng..

[3] Dhar P., Amevorku R., Amoateng-Mensah D., Sundaresan M. (2026). Deep Learning-Based Depth Estimation of Flat Bottom Holes in CFRP Using Active Infrared Thermography. SAMPE J..

[4] Berthelot J.M., Rhazi J. (1990). Acoustic emission in carbon fibre composites. Compos. Sci. Technol..

[5] De Groot P.J., Wijnen A.M., Janssen R.B.F. (1995). Real-time frequency determination of acoustic emission for different fracture mechanisms in carbon/epoxy composites. Compos. Sci. Technol..

[6] Mizutani Y., Nagashima K., Takemoto M., Ono K. (2000). Fracture mechanism characterization of cross-ply carbon-fiber composites using acoustic emission analysis. NDT E Int..

[7] Liu P.F., Chu J.K., Liu Y.L., Zheng J.Y. (2012). A study on the failure mechanisms of carbon fiber/epoxy composite laminates using acoustic emission. Mater. Des..

[8] Rijal M., Amoateng-Mensah D., Sundaresan M.J. (2024). Finite Element Simulation of Acoustic Emissions from Different Failure Mechanisms in Composite Materials. Materials.

[9] Muir C., Swaminathan B., Almansour A.S., Sevener K., Smith C., Presby M., Kiser J.D., Pollock T.M., Daly S. (2021). Damage mechanism identification in composites via machine learning and Acoustic Emission. Comput. Mater..

[10] Amevorku R.D., Amoateng-Mensah D., Rijal M., Sundaresan M.J. (2025). Classification and Clustering of Fiber Break Events in Thermoset CFRP Using Acoustic Emission and Machine Learning. Sensors.

[11] Gholizadeh S., Leman Z., Baharudin B.T.H.T. (2023). State-of-the-art ensemble learning and unsupervised learning in fatigue crack recognition of glass fiber reinforced polyester composite (GFRP) using acoustic emission. Ultrasonics.

[12] The Acoustic Emission Company (2021). AMSY-6 System Description.

[13] Ono K. (2018). Review on Structural Health Evaluation with Acoustic Emission. Appl. Sci..

[14] Šofer M., Šofer P., Pagáč M., Volodarskaja A., Babiuch M., Gruň F. (2023). Acoustic Emission Signal Characterisation of Failure Mechanisms in CFRP Composites Using Dual-Sensor Approach and Spectral Clustering Technique. Polymers.

[15] McCrory J.P., Al-Jumaili S.K., Crivelli D., Pearson M.R., Eaton M.J., Featherston C.A., Guagliano M., Holford K.M., Pullin R. (2015). Damage classification in carbon fibre composites using acoustic emission: A comparison of three techniques. Compos. Part B Eng..

[16] Marec A., Thomas J.-H., El Guerjouma R. (2008). Damage characterization of polymer-based composite materials: Multivariable analysis and wavelet transform for clustering acoustic emission data. Mech. Syst. Signal Process..

[17] Minhaj T.B., Amevorku R.D., Chaudhary B.B., Rijal M., Sundaresan M.J. Assessment of damage evolution in thermoplastic composite using acoustic emission and deep learning models. Proceedings of the SPE ACCE.

[18] Zhao Z., Chen N. (2024). Damage Localization on Wind Turbine Blades Using Acoustic Emission (AE) Signals and Graph Neural Network (GNN). Proceedings of the ASME 2024 43rd International Conference on Ocean, Offshore and Arctic Engineering.

[19] Zhang R., Cheng Y., Huang J., Zhang Y., Yan H. (2024). Unsupervised weathering identification of grottoes sandstone via statistical features of acoustic emission signals and graph neural network. Herit. Sci..

[20] Pham Q., Zemouri R.A., Shastri Y. (2024). Can graph neural networks outperform in supervised classification of noisy acoustic signals? An industrial case study of hydroelectric generator. Prognostics and System Health Management Conference (PHM).

[21] Wang J., Liu X.-Z., Ni Y.Q. (2018). A Bayesian Probabilistic Approach for Acoustic Emission-Based Rail Condition Assessment. Comput.-Aided Civ. Infrastruct. Eng..

[22] Masoud R. (2011). A Bayesian Framework for Structural Health Management Using Acoustic Emission Monitoring and Periodic Inspections.

[23] Lindley C.A., Jones M.R., Rogers T.J., Cross E.J., Dwyer-Joyce R.S., Dervilis N., Worden K. (2024). A probabilistic approach for acoustic emission based monitoring techniques: With application to structural health monitoring. Mech. Syst. Signal Process..

[24] Vaiyapuri T., Elashmawi W.H., Shridevi S., Asiedu W. (2024). VAE-CNN: Deep Learning on Small Sample Dataset Improves Hydrogen Yield Prediction in Co-gasification. J. Comput. Cogn. Eng..

[25] Sagar R.V., Basu D.J. (2023). Damage progression and crack classification in Reinforced Concrete structures under quasi-static monotonically increasing loading based on acoustic emission waveform parameters. J. Build. Eng..

[26] Olorunlambe K.A., Hua Z., Shepherd D.E.T., Dearn K.D. (2021). Towards a Diagnostic Tool for Diagnosing Joint Pathologies: Supervised Learning of Acoustic Emission Signals. Sensors.

[27] Yan J., Lee J. (2005). Degradation Assessment and Fault Modes Classification Using Logistic Regression. ASME. J. Manuf. Sci. Eng..

[28] Hamstad M.A. (1986). A review: Acoustic emission, a tool for composite-materials studies. Exp. Mech..

[29] Rose J.L. (1999). Ultrasonic Waves in Solid Media.

[30] (2021). Standard Terminology for Nondestructive Examinations.

[31] Grosse G.U., Ohtsu M. (2008). Acoustic Emission Testing.

[32] Pollock A.A. (1989). Acoustic emission inspection. Nondestructive Evaluation and Quality Control.

[33] Sause M. (2016). In Situ Monitoring of Fiber-Reinforced Composites: Theory, Basic Concepts, Methods, and Applications.

[34] Amoateng-Mensah D. (2024). Quantification of Acoustic Emission Energy Generated by Crack Growth in Thin Metallic Beams and Plates. Master’s Thesis.

[35] de Castro Saiki L.E., Gomes G.F. (2024). Understanding and Mitigating Delamination in Composite Materials: A Comprehensive Review. Mech. Adv. Mater. Struct..

[36] Carroll H.E., Matthams T.J., Davies A.J. (2003). Delamination growth during fatigue of advanced polymer matrix composites. Plast. Rubber Compos..

[37] Mohammadi R., Assaad M., Imran A., Fotouhi M. (2024). Fractographic analysis of damage mechanisms dominated by delamination in composite laminates: A comprehensive review. Polym. Test..

[38] Alfarizi M.I., Budiman B.A. (2021). The Effect of Micro Damages to Mechanical and Electrical Behaviors of Carbon Fiber Reinforced Polymer: A Review. 3rd International Symposium on Material and Electrical Engineering Conference (ISMEE), Bandung, Indonesia.

[39] Guo R., Li C., Niu Y., Xian G. (2022). The fatigue performances of carbon fiber reinforced polymer composites—A review. J. Mater. Res. Technol..

[40] Kimura M., Watanabe T., Takeichi Y., Niwa Y. (2019). Nanoscopic origin of cracks in carbon fibre-reinforced plastic composites. Sci. Rep. Sci. Rep..

[41] Li Y., Yang Z. (2025). Characterization and Simulation-Based Analysis of the Damage Evolution of Carbon Fiber Composites Under Transverse Compression. Polym. Compos..

[42] Wirtz S.F., Bach S., Söffker D. Experimental Results of Acoustic Emission Attenuation Due to Wave Propagation in Composites. Proceedings of the Annual Conference of the PHM Society.

[43] Asamene K., Hudson L., Sundaresan M. (2015). Influence of attenuation on acoustic emission signals in carbon fiber reinforced polymer panels. Ultrasonics.

[44] Eaton M., Holford K., Featherston C., Pullin R. (2007). Damage in carbon fibre composites: The discrimination of acoustic emission signals using frequency. J. Acoust. Emiss..

[45] International A. (1997). ASTM D3039: Standard Test Method for Tensile Properties of Polymer Matrix Composite Materials.

[46] Bhuiyan Y., Lin B., Giurgiutiu V. (2019). Characterization of piezoelectric wafer active sensor for acoustic emission sensing. Ultrasonics.

[47] Mills-Dadson B., Tran D., Asamene K., Whitlow T., Sundaresan M. (2017). Acoustic emission monitoring of unstable damage growth in CFRP composites under tension. 43rd Annual Review of Progress in Quantitative Nondestructive Evaluation, Atlanta.

[48] Astm E. (2021). Standard Guide for Determining the Reproducibility of Acoustic Emission Sensor Response.

[49] Ohtsu M. (1995). Acoustic Emission Theory for Moment Tensor Analysis. Res. Nondestruct. Eval..

[50] Maio L., Fromme P. (2022). On ultrasound propagation in composite laminates: Advances in numerical simulation. Prog. Aerosp. Sci..

[51] Li F., Peng H., Sun X., Wang J., Meng G. (2012). Wave propagation analysis in composite laminates containing a delamination using a three-dimensional spectral element method. Math. Probl. Eng..

[52] Nguyen M. (2025). Advanced Modeling and Data Challenges.

[53] Albert A., Anderson J.A. (1984). On the Existence of Maximum Likelihood Estimates in Logistic Regression Models. Biometrika.

[54] Heinze G., Schemper M. (2002). A solution to the problem of separation in logistic regression. Stat. Med..

[55] Firth D. (1993). Bias reduction of maximum likelihood estimates. Biometrika.

[56] Mienye I.D., Swart T.G. (2024). A Comprehensive Review of Deep Learning: Architectures, Recent Advances, and Applications. Information.

[57] Shwartz-Ziv R., Armon A. (2022). Tabular data: Deep learning is not all you need. Inf. Fusion.

[58] He X., Chen Y. (2021). Modifications of the Multi-Layer Perceptron for Hyperspectral Image Classification. Remote Sens..

